# 3D printed strontium-doped calcium phosphate ceramic scaffold enhances early angiogenesis and promotes bone repair through the regulation of macrophage polarization

**DOI:** 10.1016/j.mtbio.2023.100871

**Published:** 2023-11-19

**Authors:** Qiuju Miao, Xiaopeng Yang, Jingjing Diao, Huanwen Ding, Yan Wu, Xiangyang Ren, Jianbo Gao, Mengze Ma, Shenyu Yang

**Affiliations:** a The First Affiliated Hospital of Zhengzhou University, Zhengzhou, 450052, People's Republic of China; b School of Medicine, South China University of Technology, Guangzhou 510006, People's Republic of China; c School of Materials Science and Engineering, South China University of Technology, Guangzhou, 510641, People's Republic of China

**Keywords:** 3D printing, Strontium-doped calcium phosphate, Macrophage polarization, Early angiogenesis, Bone repair

## Abstract

The vascularization of bone repair materials is one of the key issues that urgently need to be addressed in the process of bone repair. The changes in macrophage phenotype and function play an important role in the process of vascularization, and endowing bone repair materials with immune regulatory characteristics to enhance angiogenesis is undoubtedly a new strategy to improve the effectiveness of bone repair. In order to improve the effect of tricalcium phosphate (TCP) on vascularization and bone repair, we doped strontium ions (Sr) into TCP (SrTCP) and prepared porous 3D printed SrTCP scaffolds using 3D printing technology, and studied the scaffold mediated macrophage polarization and subsequent vascularization and bone regeneration. The results of the interaction between the scaffold and macrophages showed that the SrTCP scaffold can promote the polarization of macrophages from M1 to M2 and secrete high concentrations of VEGF and PDGF-bb cytokines, which shows excellent angiogenic potential. When human umbilical vein endothelial cells (HUVECs) were co-cultured with macrophage-conditioned medium of SrTCP scaffold, HUVECs exhibited excellent early angiogenesis-promoting effects in terms of scratch healing, angiogenic gene expression, and in vitro tube formation performance. The results of in vivo bone repair experiments showed that the SrTCP scaffold formed a vascular network with high density and quantity in the bone defect area, which could increase the rate of new bone formation and advance the period of bone formation, and finally achieved a better bone repair effect. We observed a cascade effect in which Sr-doped SrTCP scaffold regulate macrophage polarization to enhance angiogenesis and promote bone repair, which may provide a new strategy for the repair of clinical bone defects.

## Graphical abstract

**Figure:**
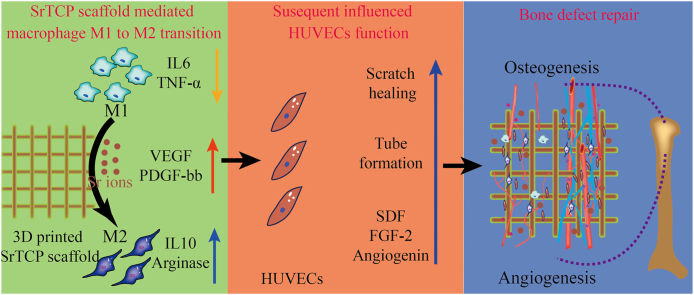

## 1 Introduction

The repair of bone defects has long been a medical problem that needs to be solved urgently, and whether bone repair materials can induce angiogenesis in the early stage and form a mature vascular network in the later stage determines the success or failure of bone defect repair [1,2]. When bone repair materials are implanted, the immune response generated by macrophages, the core of the immune system, not only produces a foreign body reaction to the bone repair materials, but also participates in and mediates the vascularization process, which is the key to the success of bone repair. Research has shown that macrophages with different phenotypes play a unique role in vascular sprouting and remodeling [[3], [4], [5], [6]]. Typically, M1 type macrophages initiate angiogenesis by secreting vascular sprouting related factors, while M2 type macrophages primarily promote vascular regeneration and remodeling by releasing vascular related cytokines [[7], [8], [9], [10]]. In recent years, the importance of angiogenesis during bone regeneration by endowing bone repair materials with immunomodulatory properties has been recognized [11,12]. Therefore, “smart” bone repair materials with immunoregulatory effects should be able to regulate the M1/M2 phenotype transformation of macrophages, release growth factors, and promote angiogenesis and bone regeneration [13,14].

Studies have shown that macrophages are not only regulated by their structural environment in the process of bone defect repair, but the biochemical environment of materials (growth factors, proteins, ions) also plays an important role in regulating the polarization phenotype of macrophages. Indispensable role [15]. Li et al. found that 3D printed calcium silicate-β-tricalcium phosphate composite scaffolds loaded with IFN-γ activated M1-M2 macrophages through the sequential release of IFN-γ and silicon, which ultimately promoted the vascularization of the scaffold [16]. Spiller et al. found that the slow release of immune regulatory factors can regulate the secretion of VEGF and PDGF-bb cytokines from M1 and M2 phenotype macrophages, which initiates the angiogenesis process and promotes the later vascular development and maturation process, thereby promoting Vascularization of bone repair materials [17,18]. Qiu et al. found a hydrogel derived from porcine acellular periosteum, which promoted the transformation of macrophage M1 to M2, angiogenesis and bone regeneration [19]. In general, the current research results show that macrophages can respond to changes in the biochemical environment of bone repair materials and release corresponding cytokines to promote the vascularization of materials, and further accelerate the repair of bone defects.

Strontium (Sr) has been proven to be a bone immune regulatory element, which can regulate immune cells to secrete osteoblast cytokines to promote osteogenesis and inhibit the activity of osteoclasts to promote rapid bone repair [[20], [21], [22]]. Guo et al. demonstrated that Sr-loaded sodium titanate nanorods can regulate the transformation of M1 macrophages to M2 macrophages by slowly releasing Sr, thereby promoting vascular formation and generation, and finally good bone formation and integration effects were observed [23]. In addition, some studies have shown that Sr has the ability to promote angiogenesis. Lu et al. found that strontium in strontium-modified titanium implants has a significant role in improving angiogenesis and regulating macrophage polarization [10]. Zhao et al. found that strontium-containing bio-glass microspheres could sequentially activate M1 and M2 macrophages through sustained release of strontium ions and Si ions, thereby effectively promoting vascularization of the scaffold [24]. Our previous studies have shown that 3D printed tricalcium phosphate (β-TCP) scaffolds have good bone repair effects [25]. However, whether Sr-doped 3D printed β-TCP scaffolds can enhance early angiogenesis and promote bone defect repair by regulating the phenotypic polarization of macrophages and their functions remains unknown. To verify this hypothesis, we doped Sr ions into β-TCP and prepared a porous SrTCP scaffold with Sr contents of 0 %, 5 %, 10 % and 20 % by 3D printing method, and used in vitro and in vivo experiments to study the scaffold-mediated macrophage polarization and subsequent vascular effects on osteogenesis and bone regeneration.

## 2 Material and methods

### 2.1 Preparation of SrTCP powder

Tricalcium phosphate (β-TCP) powder was prepared by solid-state reaction sintering method [26]. Specifically, CaHPO_4_·2H_2_O (Aladdin) and CaCO_3_ (Aladdin) were fully mixed at a molar ratio of 2.13:1 to obtain a mixed powder, and then the mixed powder was calcined at 900 °C for 4 h, and the TCP powder was obtained after cooling down to room temperature. For strontium-doped tricalcium phosphate (SrTCP), its preparation method is the same as that of β-TCP, only CaCO_3_ is replaced by SrCO_3_ (Aladdin) in proportion, and SrTCP with Sr/(Sr + Ca) ratio of 5 %, 10 % and 20 % is named Sr5TCP, Sr10 TCP and Sr20 TCP respectively.

### 2.2 Preparation of 3D printed SrTCP ceramic scaffold

According to previous research [27], 1 wt% HPMC and 1.5 wt% PAA-NH_4_ were used as thickener and dispersant for 3D printing β-TCP slurry, respectively, and the solid content of 3D printing slurry was 50w/v%. Specifically, PAA-NH_4_ was dissolved in deionized water, and its pH was adjusted to 9 with ammonia water, then β-TCP or SrTCP powder and HPMC powder were added and ball milled for 12 h, and the 3D printing slurry was obtained after ultrasonic removal of air bubbles. A 3D printing model with a diameter of 8 mm, a height of 3 mm, a wire spacing (hole size) of 400 μm and a line width of 400 μm was constructed, and then the scaffold was printed at 50 °C with a bio-3D printer (Bio-Architect X, Hangzhou, China) based on the 3D printing model. The obtained scaffold was naturally dried at room temperature for 72 h, then vacuum-dried at 50 °C for 24 h, and finally the scaffold was sintered at 1100 °C for 3 h, and the 3D printed β-TCP or SrTCP scaffold was obtained after natural cooling.

### 2.3 Characterization of SrTCP powder and 3D printed scaffold

#### 2.3.1 X-ray diffraction (XRD)

The phase of SrTCP powder and 3D printed scaffold were detected by X-ray diffractometer (XRD, Bruker AXS, Germany, D8 Advance), equipped with a Cu-Kα (λ = 1.54 Å), the voltage was 40 kV, the current was 20 mA. The XRD patterns were gathered in the range of 5° to 80° with a scanning rate of 2°/min.

#### 2.3.2 Fourier transform infrared (FTIR)

Fourier transform infrared (FT-IR) spectra of SrTCP powder was recorded using a FT-IR spectrometer (Bruker, Germany, EQUINOX55) with KBr pellets in the range of 4000–500 cm^−1^.

#### 2.3.3 Scanning electron microscopy (SEM) analysis

All samples were sputter-coated with an Ion Sputter (E−1010, Hitachi, Japan), and the crystal morphology was observed by scanning electron microscopy (SEM) (S2400, Hitachi, Japan) at 15 kV at different magnification. For the 3D printed scaffold, the spacing (pore size) between the scaffold printing wires was obtained by measuring the average value of 20 pore diameters in the SEM image by ImageJ software. EDX energy spectrum obtained by SEM EDX.

#### 2.3.4 Three-dimensional structure of scaffold

The specimens in each group were examined using a Micro-CT system (SkyScan1272, Bruker, Germany) with a spot size of 30 μm. Continuous tomographic images were obtained after scanning, and a three-dimensional reconstruction of the scanned images was carried out using the machine's proprietary software. According to the analysis and calculation of Micro-CT scanning results, the porosity of the scaffold and the connectivity rate of internal pores are calculated.

#### 2.3.5 Mechanical property analysis

The compression performance of the 3D printed scaffold was tested by a universal mechanical testing machine (Chengde Precision Testing Machine Co., Ltd, China, 10 S T), and the compression rate was 2 mm/min.

### 2.4 Interaction between 3D printing SrTCP scaffold and macrophages

#### 2.4.1 Cell culture and seeding

Raw 264.7 cells were purchased from American type culture collection (ATCC) and cultured at 37 °C in a humidified 5 % CO_2_ atmosphere with Dulbecco's modified eagle medium (DMEM, Gibco) containing 10 % fetal bovine serum (FBS, Hyclone) and 1 % streptomycin penicillin (Invitrogen). M1 macrophage activation is achieved by using LPS (100 ng/mL, Peprotech) and IFN- γ (20 ng/mL, Peprotech) to stimulate macrophages, and M2 macrophage activation was achieved by treating macrophage s with IL-4 (20 ng/mL, Peprotech) and IL-13 (10 ng/mL, Peprotech). For cell seeding, the sterilized scaffolds were soaked in medium overnight, and then the M1 or M2 macrophage suspension was evenly spread on the scaffolds, and the scaffolds were incubated at 37 °C in a humidified 5 % CO_2_ atmosphere for 1 h, and finally complete medium was added to continue the culture.

#### 2.4.2 Macrophage proliferation and adhesion on scaffolds

Macrophage proliferation: M1 or M2 macrophages were seeded on the scaffold surface at a density of 5 × 10^4^ cells/well and co-cultured for 1, 3 and 7 days. At given time intervals, the supernatant was removed and CCK-8 working solution (a mixture of 90 % complete medium and 10 % CCK-8 stock solution) was added. After additional incubation for 1 h and then 1min of slow shaking, the absorbance (OD value) of the mixed solution was measured at 450 nm by a microplate reader (BIO-RAD 680, USA).

Macrophage adhesion: After the M2 macrophage were co-cultured with the scaffold for 7 days, the original medium was discarded and washed with PBS, and then the cell live/dead working solution (Calcein-AM (2 mM, for live cell staining), PI (1.5 mM, dead cell staining)) were added and incubated in the dark for 30 min. Finally, the excess staining solution was removed and washed slowly with PBS, and the cell adhesion on the scaffold was observed using a fluorescence microscope.

#### 2.4.3 Expression of polarization-related genes in macrophages

In order to simulate the state of acute inflammatory response generated by the material at the initial stage of implantation, M1 macrophages were seeded on the surface of the scaffold, and the expression of cell polarization-related genes was detected by qPCR. To investigate the immunomodulatory function of the SrTCP scaffold, M1 macrophages were seeded on the surface of the scaffold at a density of 1 × 10^5^ cells/well. After 3 and 7 days of culture, total RNA was achieved using a Total RNA Kit (Omega, USA) according to the procedures given by the manufacturer. cDNA was prepared using HiScript®II Q RT SuperMix for qPCR (+ gDNA wiper) (Vazyme, China). Real-time qPCR was conducted using AceQ®qPCR SYBR®Green Master Mix (Vazyme, China) in a CFX96 Real-Time PCR Detection System (Bio-Rad) to analyze the gene expression of inflammatory related genes (TNF-α, IL6), anti-inflammatory related genes (IL10, Arginase), and angiogenesis related genes (Angiogenin, FGF-2 and SDF). The primers and their corresponding sequences used for the real-time qPCR analysis are listed in Table S1, and GAPDH was the control.

#### 2.4.4 Cytokine measurements

Measurement of cytokines present in cell culture supernatants, specifically TNF-α (Peprotech), TGF-β, VEGF and PDGF-bb, was conducted by ELISA according to the manufacturer's protocols.

### 2.5 Effect of macrophage paracrine on HUVECs

#### 2.5.1 Conditioned medium preparation

The conditioned medium (CM) is the medium supernatant produced by the co-culture of M1 macrophages and scaffolds. M1 Macrophages were seeded on the surface of the scaffold at a density of 2 × 10^4^ cells/well, and the culture supernatant (CM) was collected after 3 days of co-culture, centrifuged and sterilized with a 0.22 μm microporous membrane to obtain CM. The conditioned media after the co-culture of 3D printed scaffolds with different Sr doped amounts and macrophages were named CM_TCP400_, CM_Sr5TCP_, CM_Sr10TCP_ and CM_Sr20TCP_.

#### 2.5.2 Scratch assay

HUVECs were seeded in 6-well plates at a density of 1 × 10^4^ cells/well, and incubated in DMEM medium containing 10 % FBS and 1 % streptomycin penicillin until the cells reached a confluence rate of 90 %. After that, a scratch was made by a sterilized 200 μL pipette tip, and PBS solution was added to remove non-adhered cells and cell debris. Different CMs were added to the culture plate and image visualized by microscopy after 0 h, 2 h and 6 h of incubation. Image J software was used to quantitatively analyze the scratch area, and the scratch healing rate was calculated using formula 1.

(1) $$Wound\;area\left(\%\right)=\frac{\text{At}}{A0}\times 100\%$$

Where: At is the scratch area at different time points; A0 is the scratch area at 0 h.

#### 2.5.3 Expression of angiogenesis-related genes

HUVECs were seeded in a 24-well plate at a density of 1 × 10^5^ cells/well, and after the cells had adhered to the wall for 24 h, the cell culture medium was replaced with a mixed culture obtained by mixing conditioned medium and complete medium at a ratio of 1:1. After 3 days of culture, total RNA was achieved and RT-PCR wad conducted to analyze the gene expression of angiogenesis-associated gene (Angiogenin, basic fibroblast growth factor (FGF-2), stromal cell-derived factor (SDF), primers and their corresponding sequences are listed in Table S2).

#### 2.5.4 Tube formation assay

HUVECs were resuspended in conditioned medium and seed at a density of 5 × 10^3^ cells/well on to the Matrigel (Corning, USA). After 2 h and 6 h of culture, the images of tube formation of HUVECs were observed and obtained with a microscope (Olympus). The tube characteristics (Branch, mesh and length of tube) were analyzed and quantified using the Angiogenesis Analyzer toolset in ImageJ [28].

### 2.6 Animal experiment

#### 2.6.1 Surgical procedure

The animal experiments were approved by the Jinan university laboratory animal ethics committee, and followed the guidelines of the Chinese National Regulations on the Use of Laboratory Animals. A total of 30 male rabbits, 12–15 weeks old, and weighting between 2.5 and 3 kg were used in this study. The rabbits were anesthetized with a combination of 3 % pentobarbital sodium (1 mL/kg, ear vein injection) with SumianxinⅡ (0.05 mL/kg, intramuscular injection), shaved and skin disinfected. Then, an incision was made on the inner side of the tibia, the muscle and periosteum were bluntly dissected and the tibial plateau exposed, and a full-thickness 8 mm diameter bone defect was drilled at the tibial plateau using an 8 mm (outer diameter) bone ring drill. A 3D printed scaffold with a diameter of 8 mm and a thickness of 3 mm was implanted into the defect, and the incision was closed gently. After surgery, all animals were given free access to water and food, and then sprayed povidone iodine on the wound surface every day for disinfection to prevent infection within 2 weeks. At given time intervals, the rabbits were sacrificed, and the bone tissues with specimens were fixed in 4 % paraformaldehyde for 24 h.

#### 2.6.2 Micro-CT analysis

After trimming the specimen to an appropriate size, the specimen was scanned by micro-CT (SkyScan 1176, Bruker, German) with a slice thickness of 25 μm and the three-dimensional (3D) structure was obtained from the serial tomographic images of the 2D reconstruction. The bone defect area was selected as the region of interest (ROI, Region of interest) through the 3D image, and then the trabecular thickness (Tb·Th), trabecular Number (Tb·N), ratio of new bone volume to total bone defect volume (BV/TV) and bone mineral density (BMD) of the 3D printed β-TCP scaffold in the ROI area at different implantation time points was analyzed.

#### 2.6.3 Histological staining

Specimens were decalcified and embedded in paraffin, and each specimen was cut into 5 μm sections. Hematoxylin and eosin staining (H&E) and Goldner's trichrome were performed to visualize bone, cartilage, and blood vessel formation in the bone defect area. Immunohistochemical staining was used to identify CCR7 (1:50, Abcam), CD206 (1:50, Santa Cruz Biotechnology), VEGF (1:200, Abcam), PDGF-bb (1:50, Abcam), CD31 (1:200, Santa Cruz Biotechnology) at the defect area. Images were observed under a light microscopy (ZEISS, Germany).

### 2.7 Statistical analysis

All statistical analyses were carried out by using a SPSS version 10.1 software (SPSS Inc., USA). Statistically significant differences (p < 0.05) between the various groups were adjusted using the Tukey-Kramer post-hoc test. All the data are expressed as means ± standard deviation (SD).

## 3 Results and discussion

### 3.1 Characteristics of SrTCP powder

**Fig. 1:**
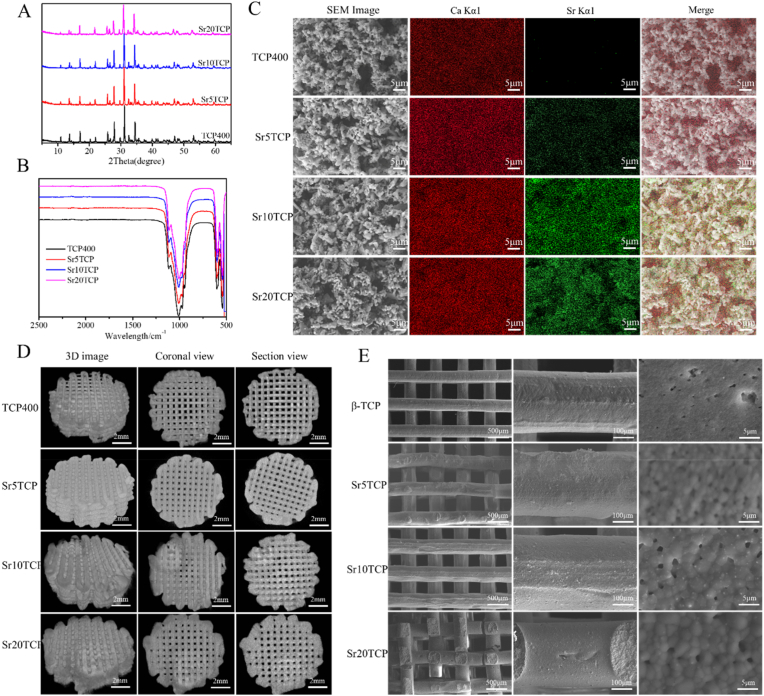
XRD (A) and FTIR (B) spectra of β-TCP and SrTCP powders with different Sr doping amounts; *C*-SEM and EDX spectrum images of β-TCP and SrTCP powder with different Sr doping content (gray is SEM image, purple is Ca EDS energy spectrum image, red is Sr EDS energy spectrum image); Micro-CT (D) and SEM(E) images of 3D printing SrTCP scaffolds with different Sr-doping amounts.

β-TCP powder is prepared by solid state sintering method, and SrTCP powder with different Sr doping amount is prepared by replacing CaCO_3_ with SrCO_3_ in proportion. XRD patterns (Fig. 1A) of the β-TCP and SrTCP with different Sr doping amount are shown in Fig. 1A. The main diffraction peaks of SrTCP correspond to the standard spectrum of β-TCP (JCPDS 46–0905), which indicates that the doping of Sr did not change the crystal form of β-TCP, which may be due to the small difference between the atomic radii of Sr and Ca (Ca-1.00 Å, Sr-1.13 Å) [29]. The FTIR spectrum of SrTCP powders with different Sr doping amounts are displayed in Fig. 1B. It can be seen that the stretching vibration peak of P=O in the PO_4-_ group is at 1020 cm^−1^, and the bending vibration peaks of P=O in the PO_4-_group are at 574 cm^−1^ and 609 cm^−1^, which shows that presence of PO_4-_ groups in SrTCP [30]. In addition, no other absorption peaks were found in the spectrogram, indicating that there were no other chemical groups in the prepared SrTCP, that is, no unexpected reactions occurred during the solid-state sintering process.

The morphology of β-TCP powder is shown in Fig. 1C. The size of the obtained SrTCP powder is between 1 and 5 μm, the particle size distribution is relatively uniform, and the surface is relatively smooth, which is extremely beneficial for the preparation of 3D printing paste. In addition, no significant difference was found in the microscopic morphology of SrTCP with different Sr doping amounts compared with β-TCP, which indicates that the Sr doping amount has little effect on the microscopic morphology of SrTCP. Furthermore, in order to determine the uniformity and distribution of Sr in the SrTCP powder, the Ca and Sr elements in the powder were measured by EDS. As the doping amount of Sr increases, the EDS intensity of Sr in SrTCP powder gradually increases, which is consistent with the results of quantitative analysis using ICP-AES (Table S1). In addition, the EDS spectra of Ca and Sr can correspond, and combined with XRD results, it can be proven that Sr partially replaces the position of Ca in the crystal.

### 3.2 Characteristics of 3D-printed SrTCP scaffolds

In order to explore the three-dimensional structure and the integrity and regularity of the internal structure of the 3D printed SrTCP scaffolds with different Sr doping amounts, Micro-CT was used to scan the SrTCP scaffolds with different Sr doping amounts, and the results are shown in Fig. 1D. The three-dimensional structures of the SrTCP scaffolds with different Sr doping amounts are relatively regular, and the coronal and cross-sectional views of the scaffolds show that the distribution of the pore structure of the scaffolds is relatively uniform, and no structural disorder and misalignment are found inside the scaffolds. The microstructure of the 3D printed SrTCP scaffold is shown in Fig. 1E. The structure of the SrTCP scaffolds with different Sr doping amounts is relatively regular, and no collapse or cracks appear during the printing process and post-processing process, and the wire diameters of the printed scaffolds are relatively uniform, which shows that the Sr did not significantly change the physical properties of β-TCP printing paste. In addition, some small pore structures were found on the surface of the scaffold, which is due to the presence of a small number of polymers in the 3D printing scaffold, such as HPMC and PAA-NH_4_, and the pore structure will eventually form with the carbonization and disappearance of the polymer during the sintering process.

In addition, combined with the SEM and Micro-CT results of the scaffold, the pore size, porosity and pore connectivity of the 3D printed scaffold were tested and analyzed (Table .1). It was found that the pore diameter, porosity and pore connectivity of the SrTCP scaffolds with different Sr doping amounts were not significantly different, which indicated that Sr did not change the pore parameters of SrTCP scaffold during printing and post-processing. This is beneficial for the subsequent study of the interaction between scaffolds and macrophages and the effect of scaffolds on bone defect repair performance with Sr doping amount as a single variable.

**Table 1:** Pore parameters of 3D printing SrTCP scaffolds with different Sr-doping amounts.

	Pre-set pore size/μm	Actual pore size/μm	Porosity/%	Pore connectivity/%
β-TCP	400	381.92 ± 17.66	62.77 ± 4.26	100 ± 0.24
Sr5TCP	400	386.64 ± 24.10	63.69 ± 5.69	99.8 ± 0.38
Sr10 TCP	400	380.64 ± 9.25	64.78 ± 3.97	99.9 ± 0.27
Sr20 TCP	400	381.18 ± 16.43	62.74 ± 4.59	100 ± 0.49

### 3.3 SrTCP scaffold regulates the polarization and function of macrophages

**Fig. 2:**
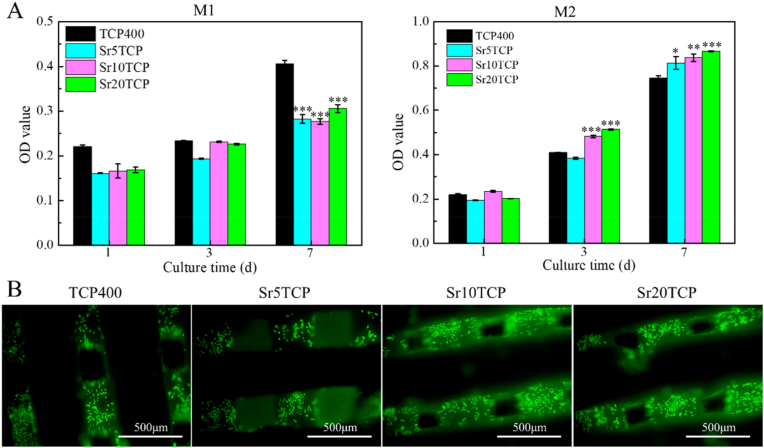
The effect of β-TCP, Sr5TCP, Sr10 TCP and Sr20 TCP scaffolds on the proliferation and adhesion of macrophages: A-OD values of M1 and M2 macrophages and scaffolds cultured for 1, 3 and 7 days, * Represents a significant difference compared to β-TCP group, *p < 0.05, **p < 0.01, ***p *<* 0.001; B-Fluorescence staining image of M2 macrophages co-cultured with the scaffold for 7 days.

Fig. 2 shows the effect of SrTCP scaffold on macrophage proliferation and adhesion. For M1 macrophages, there was no significant difference in OD values of SrTCP scaffolds with different Sr doping amounts at 1 day and 3 days, while the OD values of M1 macrophages in the SrTCP scaffold group were significantly lower than those in the β-TCP group at 7 days, which may be due to the inhibitory effect of Sr ions on the proliferation of M1 macrophages. For M2 macrophages, the OD values of SrTCP scaffolds and M2 macrophages co-cultured for 3 days and 7 days were higher than those of β-TCP group, and there was a significant difference (p < 0.05), which indicated that Sr-doped SrTCP scaffold can significantly promote the proliferation of M2 macrophages. In addition, to investigate the adhesion of macrophages on the surface of the SrTCP scaffold, M2 type macrophages co-cultured with the scaffold for 7 days were subjected to fluorescence staining, as shown in Fig. 2B. It can be found that more cells are uniformly adhered to the surface of the scaffolds with different Sr doping amounts, which not only indicates that the SrTCP scaffold has good cell compatibility, but also indicates that M2 macrophages can adhere well to the surface of the SrTCP scaffold.

**Fig. 3:**
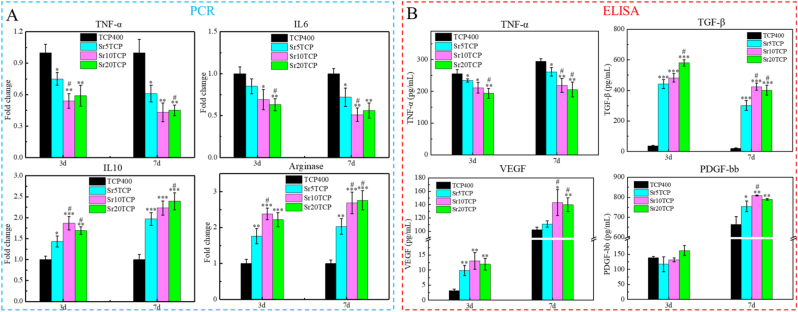
A-Inflammation-related genes expression of M1 type macrophages incubated with SrTCPSrTCP scaffold with different pore diameters for 3 and 7 days (TNF-α and IL6 are pro-inflammation related genes; IL10 and Arginase are anti-inflammation related genes). B-Changes in the concentrations of cytokines TNF-α, TGF-β, VEGF and PDGF-bb related to macrophage polarization in the supernatant after co-culture of M1 type macrophages with scaffolds for 3 and 7 days. * Represents a significant difference compared to β-TCP group, *p < 0.05, **p < 0.01, ***p < 0.001, # represents a significant difference compared to Sr5TCP group, #*p* < 0.05.

In order to simulate the state of acute inflammatory response generated by the material at the initial stage of implantation, M1 macrophages were seeded on the surface of the scaffold, and the expression of cell polarization-related genes was detected by qPCR, as shown in Fig. 3. Compared with the TCP400 group, the expressions of pro-inflammatory genes TNF-α and IL6 in the SrTCP group with different Sr doping amounts were significantly decreased, and there was a significant difference (*p* < 0.05), which indicated that the SrTCP scaffold incorporated in the SrTCP scaffold Possess immunomodulatory effects, which can inhibit the expression of pro-inflammatory genes. Contrary to the results of pro-inflammatory gene expression, the expression of anti-inflammatory genes IL10 and Arginase in the SrTCP group with different Sr doping amounts was higher than that in the β-TCP scaffold group (*p* < 0.05). Interestingly, the expression of anti-inflammatory genes is dependent on the amount of Sr doping, and the expression of anti-inflammatory genes in the Sr10 TCP and Sr20 TCP groups was significantly higher than that in the Sr5TCP group. In addition, the concentrations of macrophage polarization-related cytokines (TNF-α and TGF-β, Fig. 3B) after co-cultured with different Sr-doped scaffolds showed the same trend as the PCR results. The PCR and ELISA results indicate that Sr doping can endow β-TCP scaffold has excellent immune regulatory effects, which can promote the polarization of macrophages from the pro-inflammatory phenotype (M1) to the pro repair phenotype (M2), and the effect is particularly significant when the Sr doping amount is greater than 10 mol%.

### 3.4 SrTCP scaffold regulates the angiogenesis-promoting potential of macrophages

In order to explore the potential of SrTCP scaffolds with different Sr-doped amounts to regulate macrophages to promote angiogenesis, the concentration of the angiogenesis-related cytokines VEGF and PDGF-bb was measured, as shown in Fig. 3B. The VEGF concentrations of SrTCP groups with different Sr doping amounts were higher than β-TCP at 3 d, while the VEGF concentrations of Sr10 TCP group and Sr20 TCP scaffold group were relatively high at 7 d, which indicated that SrTCP scaffold could regulates macrophages to secrete higher concentrations of VEGF growth factor in the early stage. Compared with β-TCP group, the concentrations of PDGF-bb in different SrTCP groups were significantly higher at 7 days (*p* < 0.05). The results showed that compared with the β-TCP scaffold, the SrTCP scaffold could regulate macrophages to release higher concentrations of VEGF cytokines in the early stage, and SrTCP could promote the polarization of M1 macrophages to M2 macrophages and secrete a higher concentration of PDGF -bb cytokine, exhibits potential to stimulate angiogenesis and stabilize blood vessel growth.

### 3.5 SrTCP scaffold regulates the performance of macrophages to promote angiogenesis in vitro

**Fig. 4:**
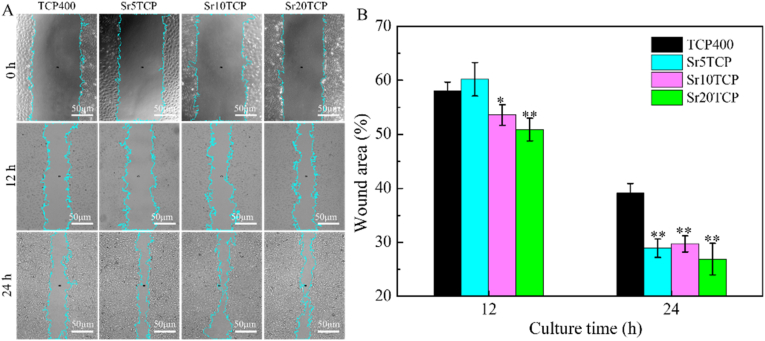
The effect of the conditioned medium of β-TCP、Sr5TCP、Sr10 TCP和Sr20 TCP scaffolds co-cultured with macrophages on the scratch healing of HUVECs: A-Optical microscopic images of the scratches of HUVECs cultured in CM for 0, 12 and 24 h; B-Quantitative analysis data of scratch healing after HUVECs cultured in conditioned medium. * Represents a significant difference between groups, *p < 0.05, **p < 0.01, ***p < 0.001.

Fig. 4 show the effect of conditioned medium after co culture of scaffold and macrophages on scratch healing of HUVECs. After 12 h and 24 h of culture, HUVECs showed more significant migration ability in the conditioned medium of the SrTCP group, and the quantitative results at 24 h showed that the scratch area of the SrTCP group with different Sr doping amounts was lower than that of the β-TCP group (*p* < 0.05), which indicated that the conditioned medium of the SrTCP group could significantly promote the migration of HUVECs and the healing of scratches.

**Fig. 5:**
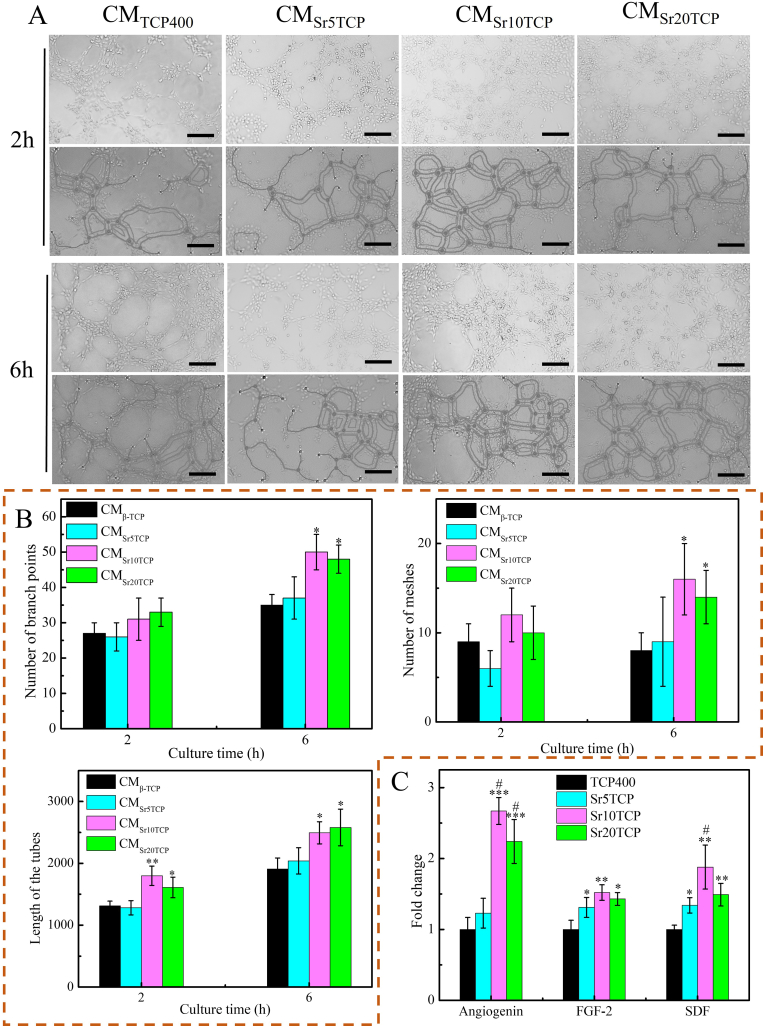
A-The structure and network image of the tube formed after HUVECs was cultured in each group of CM for 2 h and 6 h; B-Quantitative analysis data of mesh, tube length and branch point formed by HUVECs cultured in each group of CM for 2 h and 6 h; *C*-mRNA expression of angiogenesis-related genes (Angiogenin, FGF and SDF) of HUVECs cultured under conditioned medium for 3 days. * Represents a significant difference compared to Β-TCP group, *p < 0.05, **p < 0.01, ***p < 0.001, # represents a significant difference compared to Sr5TCP group #*p* < 0.05.

Fig. 5 are the images of the tube structure and network formed by HUVECs cultured in conditioned medium for 2 h and 6 h, and the quantitative data of grid number, tube length and branch point. Fig. 5A shows that the HUVECs in each group have gradually formed a tubular structure when cultured for 2 h, and the tubular structure grew, derived and formed a network structure during the period from 2 h to 6 h, among which the network structure formed by the Sr10 TCP and Sr20 TCP groups was more significant. The quantitative data results showed that (Fig. 5B) the length of the tubes formed in the SrTCP group was greater than that in the TCP400 group at 2 and 6 h, while the number of networks and branch points formed in the Sr10 TCP and Sr20 TCP groups were greater than those in TCP400 (p < 0.05), which indicated that the conditioned medium of the SrTCP scaffold group could promote the formation of rich tubular structures of HUVECs. The results of angiogenesis-related genes after HUVECs were co-cultured with conditioned medium for 3 days showed that the expression of Angiogenin (Fig. 5C) in SrTCP scaffolds with different Sr doping levels was significantly up-regulated compared with Β-TCP group, especially when the Sr doping level was 10 mol%. In addition, the results of FGF-2 and SDF gene expression also showed a similar trend to that of Angiogenin gene. The above results indicated that Sr-doped SrTCP scaffolds and CMs co-cultured with macrophages could significantly promote angiogenesis in vitro, which was closely related to the angiogenesis-associated growth factors released by macrophages.

### 3.6 In vivo animal experiments

**Fig. 6:**
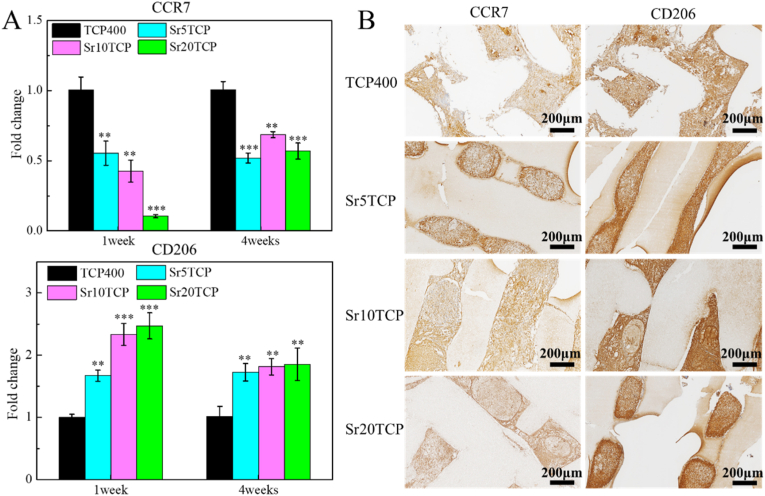
A-The expression of macrophage-related markers in the bone defect area after the scaffold was implanted into the bone defect for 1 week and 4 weeks, * represents a significant difference compared to Β-TCP group, *p < 0.05, **p < 0.01, ***p < 0.001. B-Immunohistochemical staining of CCR7 and CD206 in the bone defect area after the scaffold was implanted into the bone defect for 1 week.

Fig. 6 is the results of PCR measurement and immunohistochemical staining of macrophage-related markers CCR7 and CD206 in the bone defect area after 1 week and 4 weeks of scaffold implantation. At 1 and 4 weeks after operation, the expression of CCR7 [31], a marker of M1 macrophages in the Sr5TCP, Sr10 TCP, and Sr20 TCP groups was significantly lower than that in the β-TCP group, and the immunohistochemical staining of CCR7 at 1 week showed that the expression of CCR7 protein gradually decreased with the gradual increase of Sr doping amount, which indicated that the inflammatory response caused by Sr-doped scaffold implantation in the defect was weak or Sr ions could polarize M1 macrophages to M2 macrophages. Furthermore, the expression of M2 type macrophage marker CD206 of SrTCP scaffold was significantly higher than that of β-TCP at 1 week and 4 weeks after surgery, and immunohistochemical staining of CD206 of at 1 week after surgery showed that the color (brown) of positive expression of CD206 of SrTCP scaffold was darker than that of β-TCP, which indicated that Sr ions can promote the polarization of macrophages into the repair-promoting M2 phenotype during bone defect repair. Interestingly, the CD206 expression level of Sr10 TCP and Sr20 TCP group was higher than that of Sr5TCP group at 1 week after operation, which indicated that the doping amount of Sr could affect the degree of polarization of macrophages to M2 type.

**Fig. 7:**
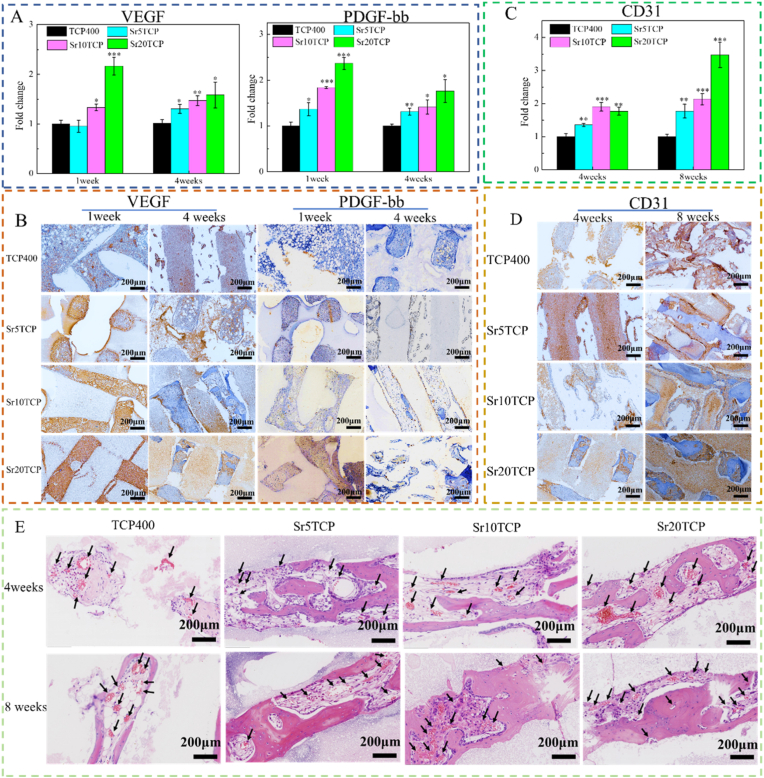
The expression of angiogenesis-related markers (A, C) and immunohistochemical staining (B, D) of the bone defect area after β-TCP、Sr5TCP、Sr10 TCP and Sr20 TCP scaffolds were implanted into the bone defect for 1 week and 4 weeks. * Represents a significant difference compared to Β-TCP group, *p < 0.05, **p < 0.01, ***p < 0.001; E-The effect of SrTCP with different strontium doping contents on micro-vessels formation: HE staining after 4 and 8 weeks of scaffold implantation (black arrows indicate the generated micro-vessels).

In order to explore the differences in angiogenesis and growth after scaffolds were implanted into bone defects, PCR assays and immunohistochemical staining were performed on the angiogenesis-related markers VEGF and PDGF-bb, and the results are shown in Fig. 7. At 1 week after operation, the expression of VEGF in Sr10 TCP and Sr20 TCP scaffolds was significantly higher than that in Β-TCP, and the results of immunohistochemical staining of VEGF also showed that the expression of VEGF protein showed a trend of increasing gradually with the increase of Sr doping amount. At 4 weeks after operation, the expression of VEGF in SrTCP scaffolds with different Sr doping amounts was higher than that in Β-TCP, which indicated that the Sr ions released from SrTCP scaffolds could promote the expression of VEGF in M1 macrophages. PDGF-bb can promote the development and maturation of blood vessels, which is necessary for angiogenesis [32]. From the PCR results and the immunohistochemical staining results of PDGF-bb, the expression of PDGF-bb in Sr5TCP, Sr10 TCP, and Sr20 TCP were higher than those of the β-TCP group at 1 and 4 weeks after operation.

CD31 is a marker of vascular endothelial tissue, which is mainly used to evaluate angiogenesis [33]. The qPCR results (Fig. 7C) showed that the expression of CD31 in the bone defect area of the Sr5TCP, Sr10 TCP, and Sr20 TCP scaffolds was significantly higher than that of the Β-TCP scaffold at 4 and 8 weeks after surgery, and the expression of CD31 was most significant at 8 weeks after surgery. Similarly, the positive expression of CD31 in the immunohistochemical staining (Fig. 7D) of CD31 at 4 and 8 weeks after surgery (brown) gradually deepened with the increase of Sr doping in the SrTCP scaffold.

**Fig. 8:**
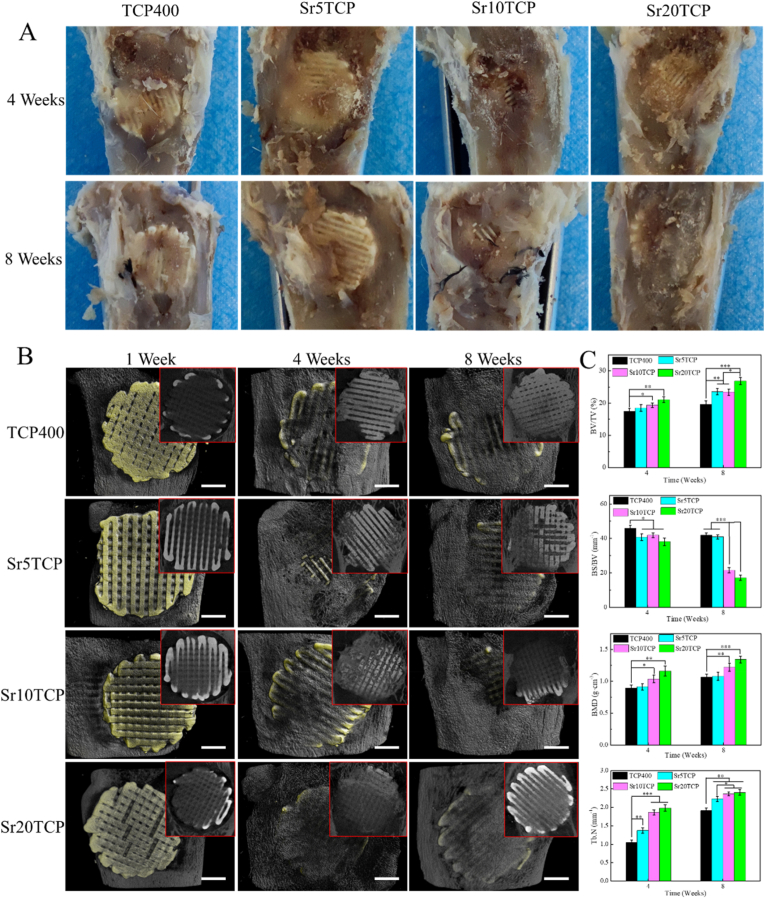
A-The macroscopic images of the TCP400, Sr5TCP, Sr10 TCP and Sr20 TCP scaffolds after the repair of the bone defect at 4 and 8 weeks after surgery; B-Micro-CT images of 1, 4, and 8 weeks after implantation of the scaffold into the bone defect (yellow is the scaffold material, the gray on the scaffold is the new bone, and the surrounding gray is the host bone, the red box is the coronal section of the micro-CT), scale bar = 2 mm; *C*-The relative bone volume (BV/TV), the trabecular thickness (Tb·Th), the number of bone trabeculae (Tb·N) and the bone mineral density (BMD) after 4 and 8 weeks of repairing bone defects with Β-TCP、Sr5TCP、Sr10 TCP and Sr20 TCP scaffolds. *, # and & represents a significant difference compared to Β-TCP, Sr5TCP and Sr10 TCP group, respectively, */#/& *p* < 0.05, **/##*p* < 0.01, ***p < 0.001.

In order to further explore the influence of the scaffold on the growth and distribution of micro-vessels, HE staining was performed on the bone tissue at 4 and 8 weeks after operation, and the results are shown in Fig. 8. The results showed that a large number of micro-vessels were found in different scaffolds at 4 weeks after operation, and the number of micro-vessels in SrTCP scaffolds was significantly higher than that in Β-TCP scaffolds. Furthermore, at 8 weeks after operation, the number of micro-vessels in each group was significantly increased compared with that at 4 weeks after operation, and the number and density of micro-vessels in SrTCP scaffolds with different Sr doping amounts were still higher than those in Β-TCP scaffold s. The results of HE staining showed that the Sr-doped SrTCP scaffold can promote the formation of micro-vessels in the process of bone defect repair, which is extremely beneficial for the rapid repair of bone defects.

Fig. 8A is the general images of TCP400, Sr5TCP, Sr10 TCP and Sr20 TCP scaffolds after they were implanted into the bone defect site for 4 weeks and 8 weeks. It can be seen that at 4 and 8 weeks after surgery, the 3D printed SrTCP scaffold maintained its original structure after implantation, without any fragmentation, collapse, or collapse, which shows that the Sr doping did not affect the strength of the scaffold. At 4 weeks after surgery, new bone callus was formed on the surface of the scaffold, and the amount of callus formation was proportional to the amount of Sr doping, which shows that Sr doping can effectively promote the formation of new bone. At 8 weeks after surgery, the amount of new callus formation increased in each group, but the increase in the Sr10 TCP and Sr20 TCP scaffold groups was more obvious, which illustrate that the Sr doped 3D printed SrTCP scaffold has better bone defect repair effects than the TCP400 scaffold.

To further explore the repair effect of the scaffold on the bone defect and the combination of the scaffold and the bone defect, Micro-CT scans were performed on the specimens after 1 week, 4 weeks and 8 weeks, as shown in Fig. 8B. At 1 week after operation, the center of the scaffolds showed varying degrees of gray, but because it was difficult to generate new bone within 1 week after operation, the gray color represented mineral deposition. It can be observed that the amount of mineral deposition in the SrTCP scaffold is significantly more than that of Β-TCP, which shows that the doping of Sr can effectively promote the deposition of minerals on the surface of the scaffold and in the defect area. At 4 weeks after operation, new bone was observed on the surface of the scaffold, but the Sr20 TCP scaffold was basically completely covered by new bone. At 8 weeks after surgery, the amount of new bone on the surface of the scaffold further increased, and the SrTCP scaffolds with different Sr doping amounts were all covered by new bone, which proved that Sr doping effectively increased the amount of 3D printed β-TCP scaffolds. Osteogenic activity, which in turn promotes the formation of new bone and the rapid repair of bone defects. In addition, it is incredible that the repair effect of Sr20 TCP scaffold on bone defect at 4 weeks is better than that of Β-TCP scaffold at 8 weeks, which further proves that the SrTCP scaffold has excellent promoting effect on new bone formation. The quantitative analysis results of Micro-CT showed that the BV/TV, Tb·Th, Tb·N, and BMD (Fig. 8C) of SrTCP scaffolds were higher than those of Β-TCP scaffolds, and there were significant differences between Sr10 TCP and Sr20 TCP compared with Β-TCP, which indicated that Sr-doped The SrTCP scaffold can form dense and large new bone tissue in the process of bone defect repair.

**Fig. 9:**
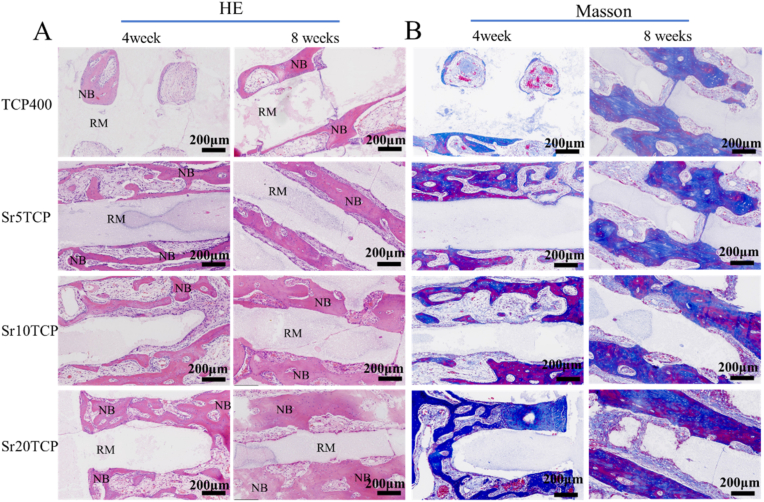
The HE (A) (NB-New bone, RM-Residual material) and Masson (B) staining images of the bone defect area after the scaffold was implanted into the bone defect 4 weeks and 8 weeks.

In order to explore the bone formation in the bone defect area, Masson and HE staining were performed on the specimens implanted into the defect for 4 and 8 weeks, as shown in Fig. 9. At 4 weeks after operation, the results of Masson staining showed that chondrocytes, cartilage transforming into bone tissue, and new bone tissue could be observed in the scaffold defect area of each group, and the bone defect area of the Sr20 TCP scaffold group formed a higher proportion new bone tissue. At 8 weeks after operation, a significant amount of new bone was formed in the defect area of each group, and as the Sr doping level gradually increased, the maturity of the new bone (dark blue) and the continuity between the new bones also increased. At 4 weeks after operation, HE staining results showed that a large number of osteoblasts grew into the scaffold and formed new bone tissue, but the new bone mass of Sr5TCP, Sr10 TCP and Sr20 TCP scaffolds was significantly higher than that of Β-TCP scaffolds. At 8 weeks after operation, continuous new bone tissue was formed in different scaffold groups, and the continuous degree of new bone tissue of SrTCP scaffold was significantly higher than that of Β-TCP scaffold, and the continuity and density of new bone of Sr20 TCP scaffold were the highest, which is consistent with the results of Micro-CT quantitative data BMD.

## 4 Discussion

The vascularization of bone repair materials is one of the key issues to be solved in the process of bone repair [19,34,35]. Changes in macrophage phenotype and function play an important role in the process of vascularization, and endowing bone repair materials with immunoregulatory properties to enhance angiogenesis is undoubtedly a new strategy to improve the effect of bone defect repair [3,6,36]. The biochemical environment of macrophages plays an integral role in regulating the polarized phenotype of macrophages. Macrophages undergo phenotypic transformation in response to changes in the biochemical environment and release corresponding cytokines to promote the rapid development of vascularization, which ultimately further accelerates the repair effect of bone defects [37,38]. Strontium (Sr) has been proven to be a bone immune regulatory element, which can regulate immune cells to secrete osteoblast cytokines and inhibit osteoclast proliferation to promote bone repair [20,39,40]. However, so far, it is still unknown whether Sr can enhance early angiogenesis and promote bone defect repair by regulating the phenotypic polarization and function of macrophages. In this study, a strontium-doped 3D-printed β-tricalcium phosphate (SrTCP) scaffold was designed and constructed, and the mechanism of whether the SrTCP scaffold can enhance early angiogenesis by regulating macrophages and ultimately promote the rapid repair of bone defects was investigated.

In this study, SrTCP powder was prepared by solid-state sintering method, and SrTCP scaffolds with different Sr doping amounts with a pore size of 400 μm were prepared by extrusion 3D technology. The results of FTIR and XRD showed that Sr replaced the position of Ca in the lattice, and the doping of Sr did not affect the phase structure and composition of SrTCP. The test results of 3D printed SrTCP scaffolds showed that the appearance structure and internal structure of 3D printed SrTCP scaffolds with different Sr doping amounts were relatively regular, and the pore diameter, porosity and pore connectivity of the scaffolds were consistent, and Sr doping did not affect the performance of the scaffolds, and the difference between the scaffolds is only reflected in the amount of Sr doping. Therefore, 3D printed SrTCP scaffolds with different Sr doping amounts were used as models to study the effects of Sr on macrophages, vascularization and bone repair.

To explore whether the 3D printed SrTCP scaffold has a regulatory effect on macrophages, ELISA and PCR were used to detect macrophage polarization-related genes and factors. PCR results revealed that SrTCP scaffolds with different Sr doping amounts exhibited a significant reduction in the expressions of pro-inflammatory genes compared with Β-TCP scaffolds. Moreover, there was a significant increase in the expression of anti-inflammatory genes (IL10 and Arginase) in SrTCP scaffolds, and this increase was dependent on the level of Sr doping. The results of ELISA showed that Sr, which has immunoregulatory effects, inhibited the secretion of pro-inflammatory related factors and promoted the secretion of pro-repair related factors. The results of ELISA and PCR indicated that Sr could endow the β-TCP scaffold with excellent immune regulation effect, which could promote the polarization of macrophages from pro-inflammatory phenotype (M1) to pro-repair phenotype (M2).

The effect of SrTCP scaffold on regulating macrophages to promote early angiogenesis was studied by the detection of blood vessel-related cytokines, PCR, scratch healing and in vitro tube formation. ELISA results showed that compared with the Β-TCP scaffold, the SrTCP scaffold could regulate macrophages to release higher concentrations of VEGF cytokines in the early stage, and SrTCP could promote the polarization of M1 macrophages to M2 macrophages and secrete higher concentrations PDGF-bb cytokine, which showed the potential to stimulate angiogenesis and stabilize blood vessel growth [18,41,42]. The results of scratch healing and PCR assays showed that the parasecretion after SrTCP co-cultured with macrophages could significantly promote the migration of HUVECs, the healing of scratches and the up-regulation of the expression of angiogenesis-related genes (Angiogenin, FGF and SDF). In vitro tube formation results showed that the conditioned medium of SRTCP could promote HUVECs to form abundant tubular structures, and the number of grids, tube length and number of branch points were significantly higher. Combining the results of expression of macrophage polarization-related genes and angiogenesis-related cytokines, it can be found that Sr doped SrTCP scaffold can regulate macrophage polarization and secrete VEGF cytokines that promote early angiogenesis and PDGF-bb cytokines that promote vascular maturation, and then HUVECs show excellent angiogenesis performance under the joint action of VEGF and PDGF-bb.

Furthermore, the in vivo angiogenesis and bone regeneration effects of SrTCP scaffolds with different Sr doping amounts were studied using the rabbit tibial defect model. The results of PCR and immunohistochemistry showed that compared with the Β-TCP scaffold, the SrTCP scaffold, especially the SrTCP scaffold with Sr doping content greater than 10 mol%, could promote the expression and secretion of VEGF by macrophages in the early stage of implantation. However, after an acute inflammatory response, the SrTCP scaffold could promote the polarization of macrophages from the M1 phenotype to the M2 phenotype and express and secrete high concentrations of PDGF-bb, which was consistent with the results of the interaction between macrophages and scaffolds in vitro [[43], [44], [45]]. The results of PCR and immunohistochemistry of CD31 and the results of HE staining confirmed that the density of micro-vessels in the bone defect area of the SrTCP scaffold group was significantly higher than that of the Β-TCP group, which is extremely beneficial for the rapid repair of bone defects [46,47]. The 3D images and quantitative results of micro-CT showed that the Sr-doped SrTCP scaffold can promote the early stage of new bone formation in the process of bone defect repair, and can promote the transformation of cartilage to new bone and increase the rate of new bone formation, which is closely related to the number of microvessels in the bone defect area.

**Fig. 10:**
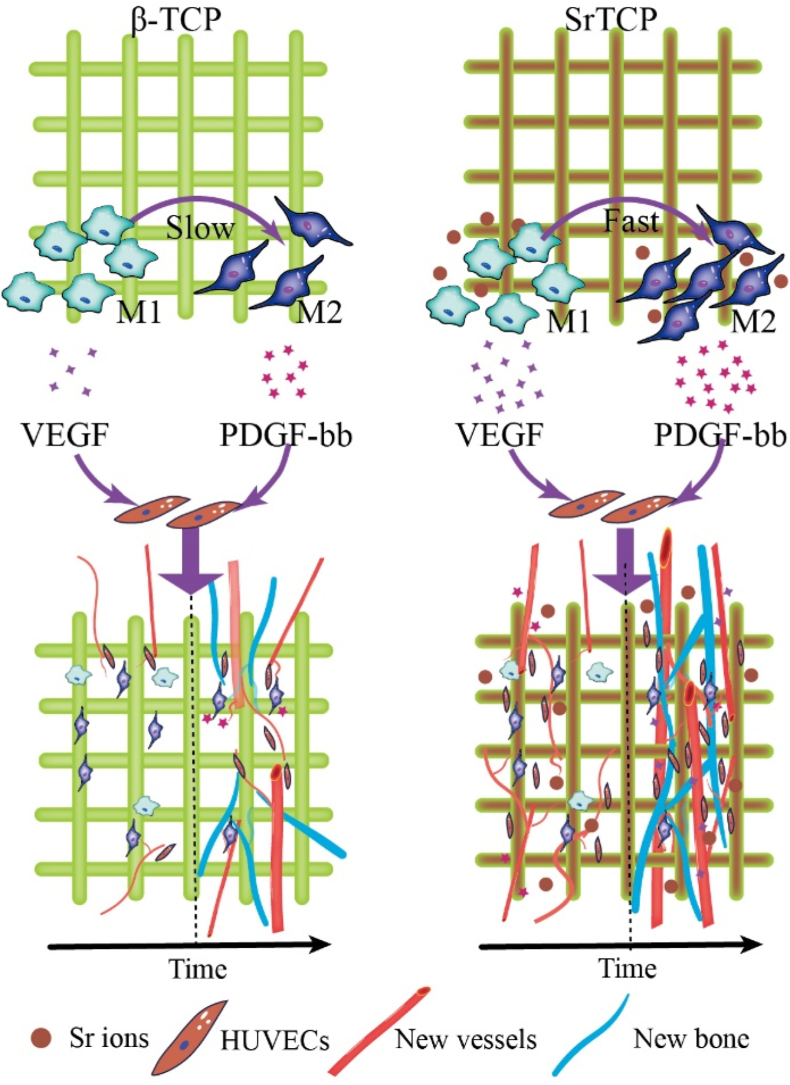
Schematic illustration of the mechanism of SrTCP scaffolds regulating macrophages to enhance vascularization and promote bone repair.

Based on the results of cell experiments and animal experiments, the mechanism of the SrTCP scaffold regulating the function of macrophages to enhance vascularization and promote bone repair can be summarized, as shown in Fig. 10. Acute inflammatory response was induced after scaffold implantation, monocyte macrophages were enriched in the defect and polarized to M1 macrophages, while M1 type macrophages released high concentrations of VEGF under the effect of Sr ions, which promoted the sprouting of blood vessels around the defect area and the growth and extension to the inside of the scaffold. After acute inflammation, due to the immunoregulatory effect of Sr, M1 macrophages are polarized into M2 macrophages under the regulation of Sr, and at the same time release high concentrations of PDGF-bb, which promotes the development of blood vessels in the defect area. The stable and high-concentration sequential secretion of VEGF and PDGF-bb promoted the ingrowth of blood vessels and the formation of microvascular network in the scaffold. Due to the coupling of angiogenesis and bone formation, the ingrowth of blood vessels is a prerequisite for bone formation, and the high density of micro-vessels has a positive impact on the rapid formation of new bone, and finally achieved a better bone defect repair effect. Although this study found the role of strontium-doped SrTCP in regulating macrophages to enhance vascularization and promote bone repair, the specific molecular mechanism is not yet clear. In future studies, we also need to explore specific molecular mechanisms such as the interaction between scaffolds and macrophages, activation of signaling pathways, release of cytokines, and regulation of macrophage polarization, which will help reveal the detailed molecular mechanism of Sr regulating macrophage polarization and provide a theoretical basis for promoting the clinical application of scaffolds.

## 5 Conclusion

3D printed SrTCP scaffolds with different Sr-doped amounts were prepared by extrusion 3D technology, and the Sr ion-mediated polarization of macrophage phenotype and its effect on subsequent angiogenesis and bone regeneration were investigated. Our data suggest that compared with the Β-TCP scaffold, the Sr-doped SrTCP scaffold can promote the polarization of macrophages from M1 to M2, and can regulate macrophages to secrete high concentrations of VEGF and PDGF-bb cytokines, thereby expressing excellent performance in promoting early angiogenesis. In addition, the SrTCP scaffold formed a vascular network with high density and quantity in the bone defect area, and promoted the transformation of cartilage to new bone and increased the rate of new bone formation, which made the new bone formation advance and finally got a better result of bone repair effect. This study explored the potential relationship among 3D printed SrTCP scaffolds, macrophage function, vascularization, and bone repair, and observed the cascade effect of bone repair materials regulating macrophage polarization to enhance angiogenesis and promote bone repair, which may provide a new strategy for rapid repair of bone defects.

## CrediT author statement

Qiuju Miao: Conceptualization, Methodology, Investigation, Writing - Original Draft; Xiaopeng Yang: Formal analysis, Investigation, Writing - Review & Editing, Funding acquisition; Jun Wang: Writing - Review & Editing; Huanwen Ding: Software, Formal analysis, Funding acquisition; Jingjing Diao: Investigation, Resources, Writing - Review & Editing, Project administration; Xiangyang Ren: Software, Formal analysis, Data Curation, Funding acquisition; Yan Wu: Validation, Writing - Review & Editing, Funding acquisition; Jianbo Gao: Writing - Review & Editing, Supervision; Mengze Ma: Methodology, Validation, Data Curation, Supervision; Shenyu Yang: Conceptualization, Methodology, Formal analysis, Data Curation, Writing - Review & Editing, Supervision, Project administration, Funding acquisition.

## Declaration of competing interest

The authors declare that they have no known competing financial interests or personal relationships that could have appeared to influence the work reported in this paper.

## Data Availability

Data will be made available on request.
