# Supplementary material for: 3D printed strontium-doped calcium phosphate ceramic scaffold enhances early angiogenesis and promotes bone repair through the regulation of macrophage polarization
Source: Mater Today Bio. 2023 Nov 19;23:100871. doi: 10.1016/j.mtbio.2023.100871 (PMC10765239; doi:10.1016/j.mtbio.2023.100871)
Supplement: Multimedia component 1 [file mmc1.docx]

**Supplementary Material**

**3D printed strontium-doped calcium phosphate ceramic scaffold enhances early angiogenesis and promotes bone repair through the regulation of macrophage polarization**

Qiuju Miao^1^, Huanwen Ding^2^, Jingjing Diao^3^, Xiangyang Ren^1^, Yan Wu^1^, Xiaopeng Yang^1^, Jianbo Gao1, Mengze Ma^1,*^, Shenyu Yang^1,*^

1The First Affiliated Hospital of Zhengzhou University, Zhengzhou, 450052, People’s Republic of China

2 School of Medicine, South China University of Technology, Guangzhou 510006, People’s Republic of China

3 School of Materials Science and Engineering, South China University of Technology, Guangzhou, 510641, China

*Corresponding author: Yang Shenyu ([ysy910724@163.com](mailto:ysy910724@163.com))

**Supplementary Figures**


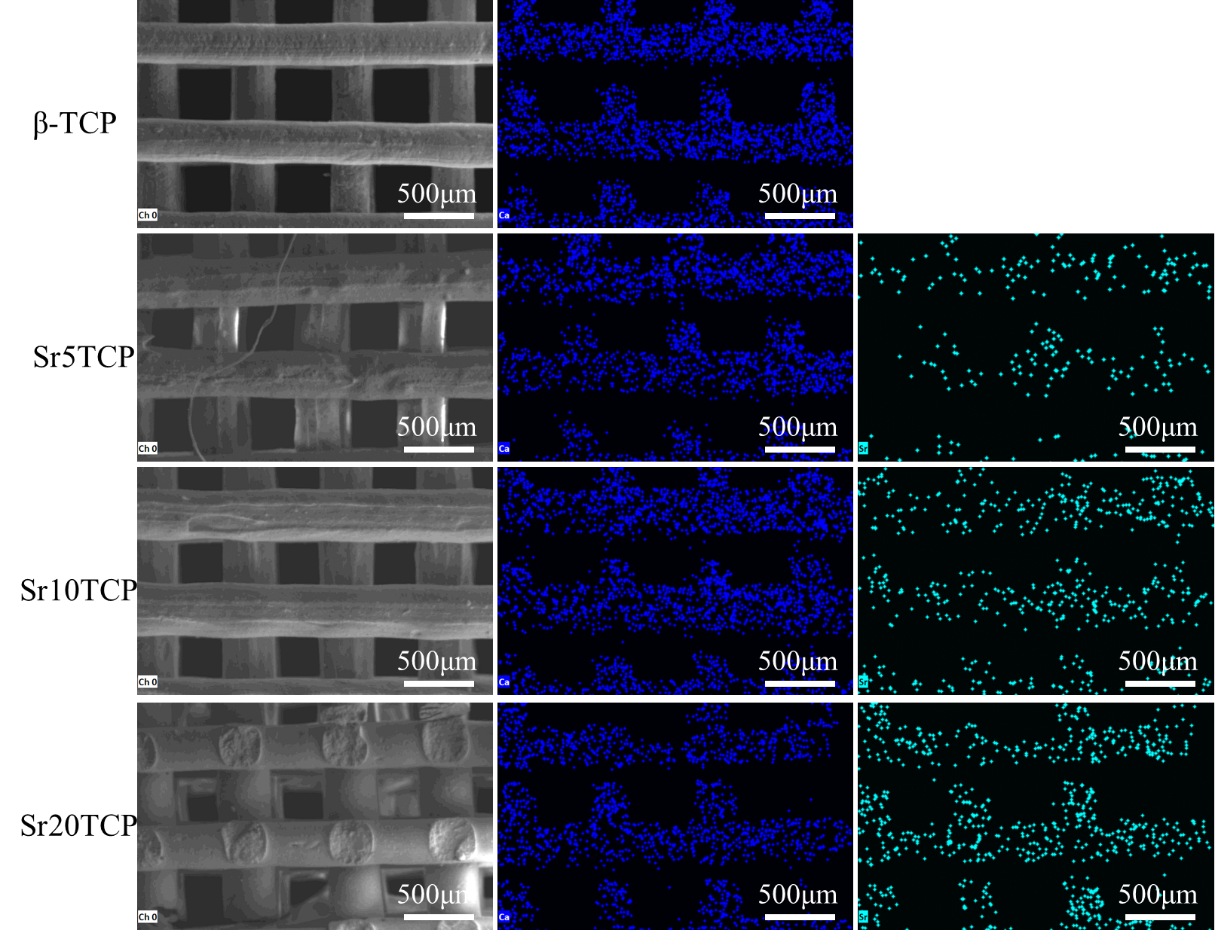


Fig.S1 EDX spectra of 3D printed SrTCP scaffolds with different Sr-doping amounts (gray is SEM image, blue is Ca, green is Sr)

Fig.S2 Compressive strength of 3D printed SrTCP scaffolds with different Sr doping amounts

Tab.S1 Sr content in SrTCP powder and SrTCP scaffold

| Names | Preset Sr doping  amount /% | Actual Sr  doping amount /% | Actual Sr doping amount in  SrTCP scaffold /% |  |
| --- | --- | --- | --- | --- |
| TCP400 | 0 | — | — |  |
| Sr5TCP | 5 | 4.95±0.08 | 4.97±0.07 |  |
| Sr10TCP | 10 | 9.88±0.11 | 9.85±0.13 |  |
| Sr20TCP | 20 | 19.82±0.12 | 19.85±0.16 |  |


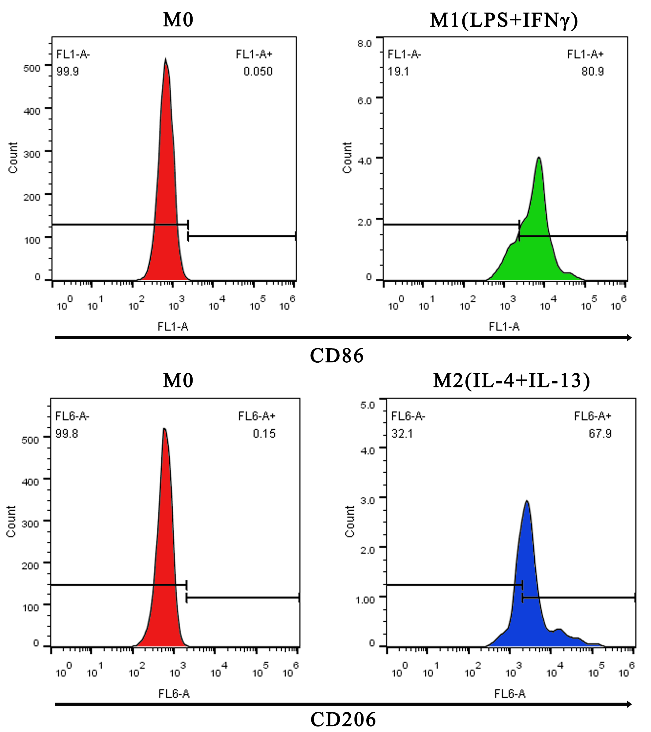


Fig.S3 Express of CD86 and CD206 detected by flow cytometry


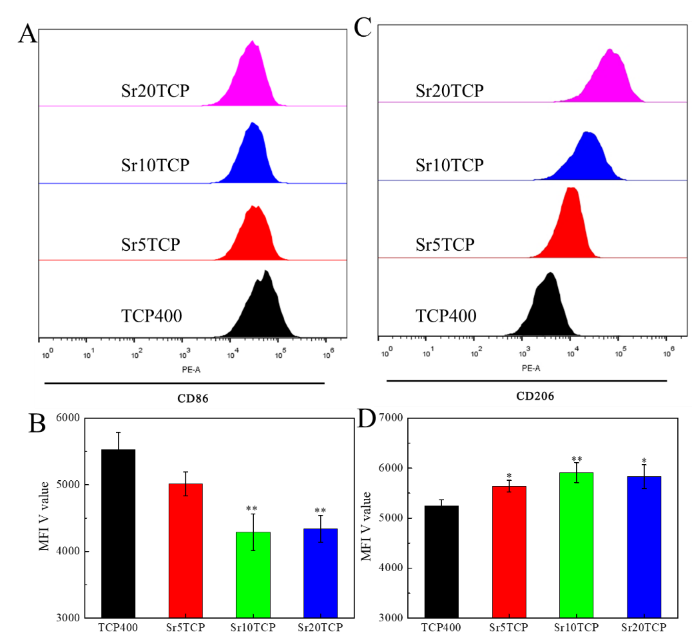


Figure S4 A-Expression of CD86 detected by flow cytometry; B-The mean fluorescence intensity of CD86; C-Expression of CD206 detected by flow cytometry; D-The mean fluorescence intensity of CD206, *p<0.05 and **p<0.01 indicate significant differences when compared to the TCP400.


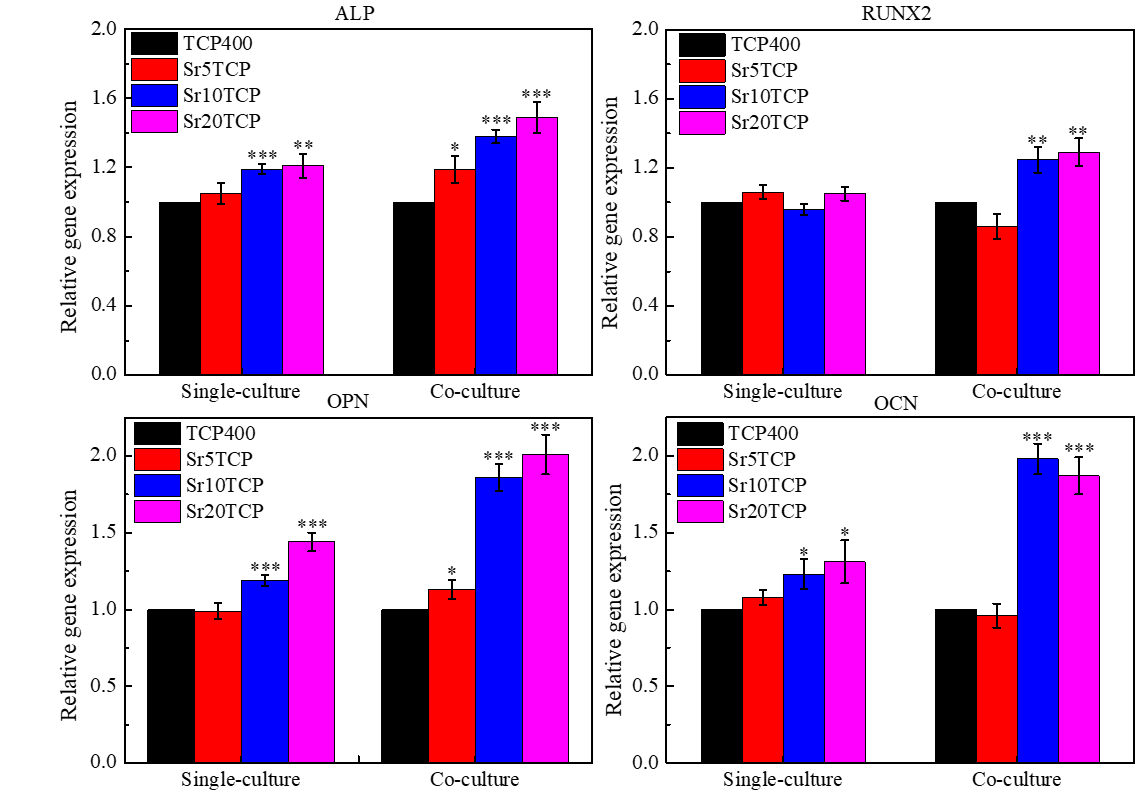


Fig.S5 The expression of osteogenesis-related genes (ALP, OCN, OPN and RUNX2) after BMSCs were cultured with scaffolds alone and in conditioned medium for 14 days, **p*<0.05, ***p*<0.01, ****p*<0.001

Tab.S2 Osteogenesis-related gene primer pairs used in the RT-qPCR

| Gene | Primer | Sequence (5’-3’) |
| --- | --- | --- |
| GAPDH | Forward | ACAGTTGCCATGTAGACC |
|  | Reverse | TTTTTGGTTGAGCACAGG |
| ALP | Forward | TCTTCACATTTGGTGGATAC |
|  | Reverse | ATGGAGACATTCTCTCGTTC |
| OCN | Forward | TTCTTTCCTCTTCCCCTTG |
|  | Reverse | CCTCTTCTGGAGTTTATTTGG |
| OPN | Forward | GACCAAGGAAAACTCACTAC |
|  | Reverse | CTGTTTAACTGGTATGGCAC |
| RUNX2 | Forward | AAGCTTGATGACTCTAAACC |
|  | Reverse | TCTGTAATCTGACTCTGTCC |


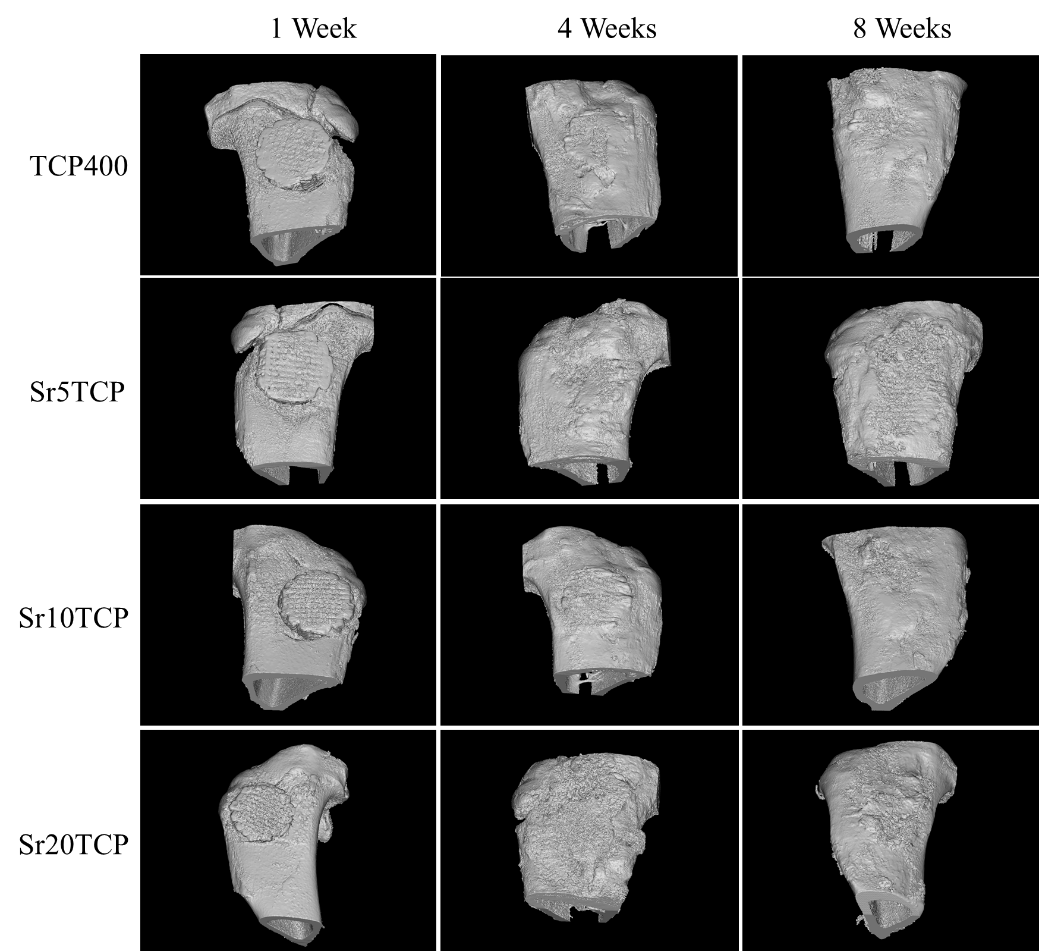


Fig.S6 Micro-CT 3D images of 1, 4, and 8 weeks after implantation of the scaffold into the bone defect

Tab.S3 Macrophage polarization related gene primer pairs used in the RT-qPCR

| Genes | Sequences of primer (5′−3′) |
| --- | --- |
| TNF-α | Forward:5'-CTGAACTTCGGGGTGATCGG-3’ |
|  | Reverse:5'-GGCTTGTCACTCGAATTTTGAGA-3’ |
| IL6 | Forward:5'-ATAGTCCTTCCTACCCCAATTTCC-3' |
|  | Reverse:5'-GATGAATTGGATGGTCTTGGTCC-3' |
| IL10 | Forward:5'-GAGAAGCATGGCCCAGAAATC-3' |
|  | Reverse:5'-GAGAAATCGATGACAGCGCC-3' |
| Arginase | Forward:5'-CATATCTGCCAAGGACATCG-3' |
|  | Reverse:5'-GGTCTCTTCCATCACTTTGC-3' |

Tab.S4 Angiogenesis related gene primer pairs used in the RT-qPCR

| Genes | Sequences of primer (5′−3′) |
| --- | --- |
| Angiogenin | Forward: 5’-GTGCTGGGTCTGGGTCTGAC-3’ |
|  | Reverse: 5’-GGCCTTGATGCTGCGCTTG-3’ |
| FGF | Forward: 5’-CTGTACTGCAAAAACGGG-3’ |
|  | Reverse: 5’-AAAGTATAGCTTTCTGCC-3’ |
| SDF | Forward: 5’-TGAGAGCTCGCTTTGAGTGA-3’ |
|  | Reverse: 5’-CACCAGGACCTTCTGTGGAT-3’ |
| GAPDH | Forward: 5’-GGAGTCCACTGGCGTCTTC-3’ |
|  | Reverse: 5’-GCTGATGATCTTGAGGCTGTTG-3’ |


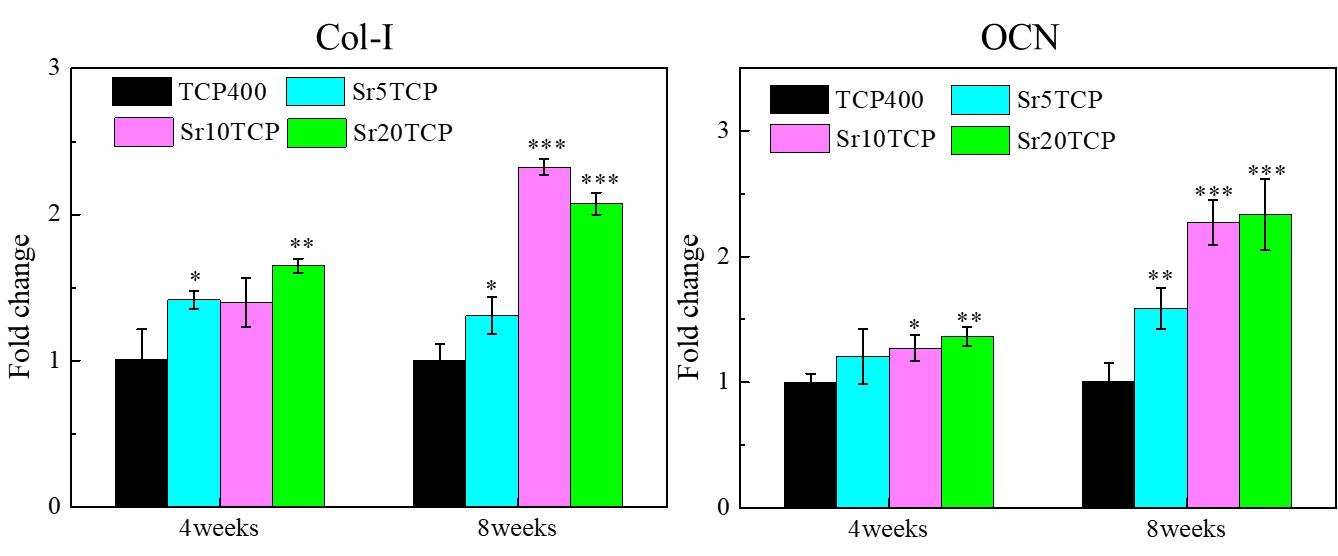


Fig.S7 The expression of the osteogenic markers Col-I and OCN of TCP400、Sr5TCP、Sr10TCP and Sr20TCP scaffolds at 4 and 8 weeks after surgery. *Represents a significant difference compared with the TCP400 group, **p*<0.05, ***p*<0.01, ****p*<0.001


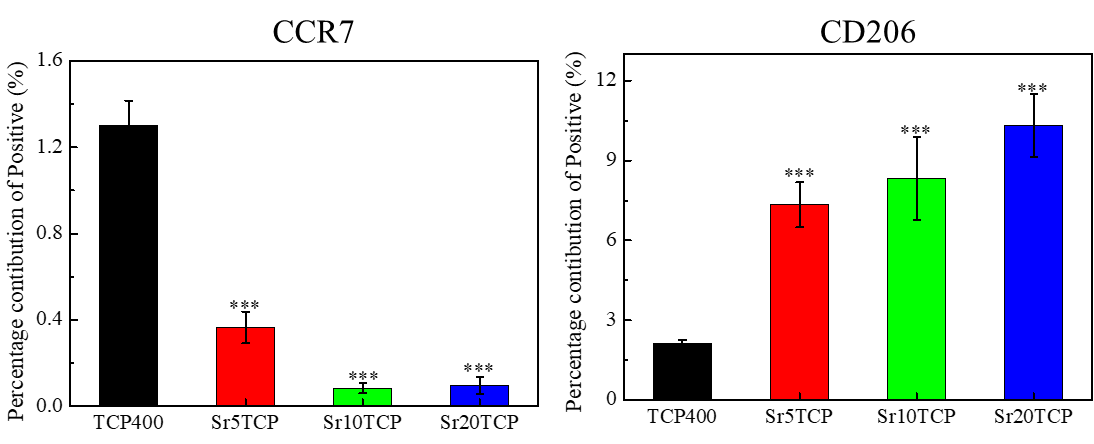


Fig.S8 Quantitative analysis results of immunohistochemical staining of CCR7 and CD206 in the bone defect area after the scaffold was implanted for 1 week, **p*<0.05, ***p*<0.01, ****p*<0.001


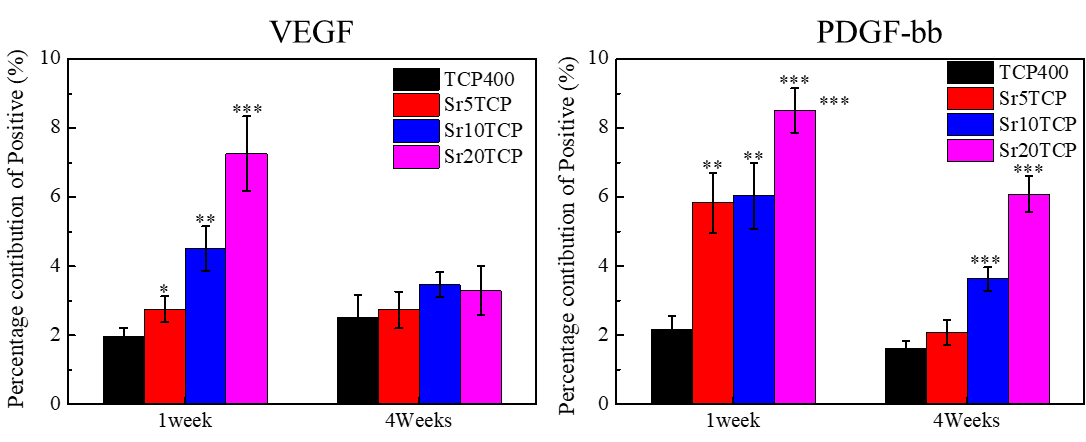


Fig.S9 Quantitative analysis results of immunohistochemical staining of VEGF and PDGF-bb in the bone defect area after the scaffold was implanted for 1 week and 4 weeks, **p*<0.05, ***p*<0.01, ****p*<0.001

Fig.S10 Quantitative analysis results of immunohistochemical staining of CD31 in the bone defect area after the scaffold was implanted for 1 week and 4 weeks, **p*<0.05, ***p*<0.01, ****p*<0.001
